# AllergoOncology: Microbiota in allergy and cancer—A European Academy for Allergy and Clinical Immunology position paper

**DOI:** 10.1111/all.13718

**Published:** 2019-03-06

**Authors:** Eva Untersmayr, Heather J. Bax, Christoph Bergmann, Rodolfo Bianchini, Wendy Cozen, Hannah J. Gould, Karin Hartmann, Debra H. Josephs, Francesca Levi‐Schaffer, Manuel L. Penichet, Liam O'Mahony, Aurelie Poli, Frank A. Redegeld, Franziska Roth‐Walter, Michelle C. Turner, Luca Vangelista, Sophia N. Karagiannis, Erika Jensen‐Jarolim

**Affiliations:** ^1^ Institute of Pathophysiology and Allergy Research Center of Pathophysiology, Infectiology and Immunology Medical University Vienna Vienna Austria; ^2^ St. John's Institute of Dermatology School of Basic & Medical Biosciences King's College London Guy's Hospital London UK; ^3^ School of Cancer and Pharmaceutical Sciences King's College London Guy's Hospital London UK; ^4^ ENT Research Institute for Clinical Studies Essen Germany; ^5^ Comparative Medicine The Interuniversity Messerli Research Institute University of Veterinary Medicine Vienna Medical University Vienna University Vienna Vienna Austria; ^6^ Center for Genetic Epidemiology Department of Preventive Medicine Keck School of Medicine of University of Southern California Los Angeles California USA; ^7^ Department of Pathology Keck School of Medicine of University of Southern California Los Angeles California USA; ^8^ Norris Comprehensive Cancer Center Keck School of Medicine of Los Angeles Los Angeles California USA; ^9^ Randall Centre for Cell and Molecular Biophysics School of Basic & Medical Biosciences King's College London New Hunt's House London UK; ^10^ Medical Research Council & Asthma UK Centre in Allergic Mechanisms of Asthma London UK; ^11^ Department of Dermatology University of Luebeck Luebeck Germany; ^12^ Pharmacology and Experimental Therapeutics Unit School of Pharmacy Faculty of Medicine The Institute for Drug Research The Hebrew University of Jerusalem Jerusalem Israel; ^13^ Division of Surgical Oncology Department of Surgery David Geffen School of Medicine University of California, Los Angeles California USA; ^14^ Department of Microbiology, Immunology and Molecular Genetics David Geffen School of Medicine University of California, Los Angeles California USA; ^15^ Jonsson Comprehensive Cancer Center University of California Los Angeles California USA; ^16^ The Molecular Biology Institute University of California Los Angeles California USA; ^17^ UCLA AIDS Institute Los Angeles California USA; ^18^ Departments of Medicine and Microbiology APC Microbiome Ireland National University of Ireland Cork Ireland; ^19^ Department of Infection and Immunity Luxembourg Institute of Health Esch‐sur‐Alzette Luxembourg; ^20^ Division of Pharmacology Faculty of Science Utrecht Institute for Pharmaceutical Sciences Utrecht University Utrecht The Netherlands; ^21^ Barcelona Institute for Global Health (ISGlobal) Barcelona Spain; ^22^ Universitat Pompeu Fabra (UPF) Barcelona Spain; ^23^ CIBER Epidemiología y Salud Pública (CIBERESP) Madrid Spain; ^24^ McLaughlin Centre for Population Health Risk Assessment University of Ottawa Ottawa Ontario Canada; ^25^ Department of Biomedical Sciences Nazarbayev University School of Medicine Astana Kazakhstan

**Keywords:** allergy, cancer, hygiene hypothesis, microbiota, oncoimmunology

## Abstract

The microbiota can play important roles in the development of human immunity and the establishment of immune homeostasis. Lifestyle factors including diet, hygiene, and exposure to viruses or bacteria, and medical interventions with antibiotics or anti‐ulcer medications, regulate phylogenetic variability and the quality of cross talk between innate and adaptive immune cells via mucosal and skin epithelia. More recently, microbiota and their composition have been linked to protective effects for health. Imbalance, however, has been linked to immune‐related diseases such as allergy and cancer, characterized by impaired, or exaggerated immune tolerance, respectively. In this AllergoOncology position paper, we focus on the increasing evidence defining the microbiota composition as a key determinant of immunity and immune tolerance, linked to the risk for the development of allergic and malignant diseases. We discuss novel insights into the role of microbiota in disease and patient responses to treatments in cancer and in allergy. These may highlight opportunities to improve patient outcomes with medical interventions supported through a restored microbiome.

**Abbreviations**
ACT: adoptive T‐cell therapy
AD: atopic dermatitis
APC: antigen presenting cells
APRIL: A proliferation‐inducing ligand
BAFF: B‐cell activating factor
CCL2: gene of C‐C motif chemokine 2
CNS: central nervous system
CTLA4: cytotoxic T lymphocyte‐associated protein 4
DC: dendritic cells
FFAR3: free fatty acid receptor 3
FOS: fructooligosaccharides
GM‐CSF: granulocyte‐macrophage colony‐stimulating factor
GMP: glycomacropeptide
GOS: galacto‐oligosaccharides
GPR: G protein–coupled receptor
HDAC: histone deacetylases
HDAC: histone deacetylases
HIV: human immunodeficiency virus
ILC: innate lymphoid cells
IL: interleukin
LBP: LPS‐binding protein
LPS: lipopolysaccharide
MAPK: mitogen‐activated protein kinase 1
NDO: non‐digestible oligosaccharides
NHL: non‐Hodgkin lymphoma
Nod‐like: nucleotide‐binding oligomerization domain‐like
PD‐1: programmed cell death protein 1
PPAR: peroxisome proliferator‐activated receptors
PRR: pattern recognition receptors
PTEN: phosphatidylinositol 3,4,5‐trisphosphate
PTP: protein tyrosine phosphatase
QS: quorum sensing
ROS: reactive oxygen species
sCD14: soluble CD14
SCFAs: short‐chain fatty acids
Th2: T helper type 2
TLR: Toll‐like receptor
TNFα: tumor necrosis factor
Treg: T regulatory cell

**Highlights**

- Microbiota composition has been linked to health protective effects representing a key determinant of immunity and immune tolerance.
- Microbiota imbalance is increasingly recognized to be associated with an enhanced risk for immune‐related diseases such as allergy and cancer.
- Novel insights into the role of microbiota in disease and patient responses to cancer or allergy treatments highlight opportunities to improve patient outcomes through a restored microbiome.

## 1 INTRODUCTION

### 1.1 Why studying microbiota is important for the field of AllergoOncology

The collective genome of *all* microorganisms living in and on the surfaces of the human body is defined as the *microbiome* and contains 150 times more genes than the 23 000 protein‐coding genes of human origin (Table 1). The human microbiome project^1^ has contributed to understanding of the composition, function, and diversity of the human *microbiota,* that is, all microorganisms populating the inner and outer surfaces of the human body, including viruses, fungi, protozoa, archaea, and bacteria (Table 2).

**Table 1:** Definitions

Term	Definition
Microbiota	Microorganisms (bacteria, viruses, fungi, protozoa, and archaea) populating the inner and outer surfaces of the human body
Microbiome	Collective genome of all microorganisms
Mutualism	A relationship between two organisms of different species resulting in benefits for both organisms from the interaction
Symbionts	Two different organisms of the same or of different species with a close and persistent biological interaction

**Table 2:** Prevalent bacteria at different sites in healthy subjects

	Phylum	Class	Order	Family	Species
Skin	Actinobacteria	Actinobacteria	Actinomycetales	Propionibacteriaceae	Propionibacterium acnes
	Firmicutes	Bacilli	Bacillales	Staphylococcaceae	Staphylococcus epidermis
Gut					
Upper part	Firmicutes	Bacilli	Lactobacillales	Streptococcaceae	Streptococcus spp.
				Lactobacillaceae	Lactobacilli spp.
Distal part	Firmicutes	Clostridia	Clostridiales	Clostridiaceae	Clostridium spp
	Actinobacteria	Actinobacteria	Bifidobacteriales	Bifidobacteriaceae	Bifidobacterium spp.
	Bacteroidetes	Bacteroidia	Bacteroidales	Bacteroidaceae	Bacteroides spp.
Lung	Bacteroidetes	Bacteroidia	Bacteroidales	Prevotellaceae	Prevotella spp.
	Firmicutes	Bacilli	Lactobacillales	Streptococcaceae	Streptococcus spp.
	Proteobacteria	Gammaproteobacteria	Pasteurellales	Pasteurellaceae	Actinobacillus spp., etc.
Nose	Actinobacteria	Actinobacteria	Actinomycetales	Corynebacteriaceae	Corynebacterium spp.
	Firmicutes	Bacilli	Bacillales	Staphylococcaceae	Staphylococcus spp.

The human microbiota show remarkable variability and have a mutualistic relationship with the human host. The microbiome profoundly affects the epithelium and the mucosal immune system and vice versa. Thereis growing evidence that microbiota play a paramount role in the control of immune‐mediated diseases such as allergy and cancer, the two complementary diseases in the frame of AllergoOncology.^2^

### 1.2 Hygiene hypothesis in allergy: from epidemiology to mechanisms

The allergy epidemics correlate with improved hygiene practices associated with urban lifestyles (Table S1). Milestone studies revealed that this epidemic can be counter‐regulated in part by exposure to a traditional farming environment. The cornerstones of the allergy protective farm effect are (a) farm activities during pregnancy, (b) early life farm exposure, and (c) raw milk consumption, but farms are also protective against viral infections. Protection depends on the farm dust levels in an area and depends on the type of farming. To give an example, children from Amish traditional pre‐industrial farming communities were less prone to allergy development than Hutterites who use modern industrial farming methods. Amish farm dust initiated innate immune pathways associated with protection from allergy development in a mouse model. Additionally, rich microbial exposure by cohabitation with wild mice improves the immune response in laboratory mice. In line with this, dog keeping is reported to protect children from asthma. Also helminth infections can support the establishment of protective microbiota (for summary and references, see Table S1).

Molecular mechanisms are increasingly understood. Viral antigens due to molecular mimicry counteract specific allergen sensitization and induce specific T effector memory responses. Exposure to N‐glycolylneuraminic acid (Neu5Gc), a sialic acid compound in farm dust, induces regulatory pathways. Endotoxin exposure of bronchial epithelial cells counteracts allergen‐induced Th2 responses. Prenatal farm exposure supports Treg and Th17 cell differentiation. The protective immunity associated with the consumption of raw milk induces FOXP3 demethylation and T regulatory cells (Tregs). Exposure of bronchial epithelia to farm dust, for instance containing CpG‐DNA, or of gut epithelia to farm milk enhances epithelial barrier integrity, resulting in protective innate immunity to allergens and viruses (Table S1).

In accordance, immature gut microbial composition at age 1 year was positively associated with asthma risk at 5 years in children with asthmatic mothers.^3^ There were inverse associations with relative abundances of genera *Faecalibacterium*,* Bifidobacterium*,* Roseburia*,* Alistipes*,* Lachnospiraceae incertae sedis*,* Ruminococcus*, and *Dialister* and a positive association with *Veillonella*. Inverse associations of relative abundances of genera *Lachnospira*,* Veillonella*,* Faecalibacterium*, and *Rothia* at age 3 months and atopy and wheeze at age 1 year were observed in another study,^4^ as was amelioration of lung inflammation in adult offspring of germ‐free mice inoculated with these taxa. The group at highest risk for multisensitized atopy (2‐years) and asthma (4‐years) was characterized by lower abundance of gut bacteria *Bifidobacterium*,* Akkermansia*,* Faecalibacterium*, and *Lactobacillus* and higher abundance of fungi *Candida* and *Rhodotorula* at 1‐11 months.^5^ Decreased gut microbiome diversity was correlated with CD4+ T‐cell decline in immune‐deficient patients.^6^ Increasing use of antibiotics has been linked with dysbiosis and enhanced prevalence of allergies and asthma.^7^ Also, the pharmacologic impairment of gastric digestion is associated with gut dysbiosis and has been correlated with allergy in mice and humans.^8^, ^9^ However, study results of probiotic supplementation in childhood asthma or wheeze are inconclusive.^10^

### 1.3 Hygiene hypothesis in oncology: from epidemiology to mechanisms

Increased incidence of certain cancers in Westernized countries^11^ may be associated with under‐exposure to certain microbial species, modern lifestyle, and consumption of sterilized food.^12^ Higher socioeconomic status is associated with increased incidence of Hodgkin lymphoma,^13^ while daycare attendance and higher number of childhood infections are linked with lower risk of acute lymphoblastic leukemia^14^ and adult chronic lymphoid leukemia.^15^ Increased cancer risk is observed in patients with autoimmune diseases and chronic allergic disorders.^16^ Mice with rapid melanoma growth and poor immunosurveillance, exhibited relatively low levels of *Bifidobacterium* species which, when restored by oral feeding and co‐housing, resulted in enhanced tumor antigen presentation and reduced malignant growth.^17^

Epidemiological studies have also examined specific bacteria, viruses, periodontal disease, and circulating antibodies to selected pathogens in relation to cancer risk.^18^ Carriage of *Porphyromonas gingivalis* and *Aggregatibacter actinomycetemcomitans* in prediagnostic oral wash samples was positively associated with pancreatic cancer risk, and *Fusobacteria* and its genus *Leptotrichia* were inversely associated.^19^ *Tannerella forsythia* was positively associated with esophageal adenocarcinoma and *Porphyromonas gingivalis* with squamous cell carcinoma.^20^ Greater abundance of genera *Corynebacterium* and *Kingella* was inversely associated with head and neck squamous cell cancer.^21^

An omnivore, but not a vegetarian, diet promotes butyrate‐producing *Lachnospiraceae* (Firmicutes/Clostridiales). *Bifidobacterium spp* (Actinobacteria/Bifidobacteriales/Bifidobacteriaceae) are strongly stimulated by dietary intake of oligosaccharides as present in milk, while *Erysipelotrichi* (Firmicutes/Erysipelotrichales/Erysipelotrichaceae) are stimulated by high fat, Western diets.^22^ A prudent diet (rich in whole grains and fiber) was associated with a lower risk of *Fusobacterium nucleatum*‐positive colorectal cancer incidence.^23^ African Americans fed a high‐fiber, low‐fat diet and rural Africans fed a high‐fat, low‐fiber diet, demonstrated reciprocal changes in colonic mucosal inflammation and biomarkers of cancer risk and in colonic microbiota and metabolome including saccharolytic fermentation and butyrogenesis, and secondary bile acid synthesis.^24^

It has also been postulated that the use of antibiotics may support cancer progression through subversion of immunosurveillance. In a transgenic mouse model of spontaneous mammary carcinoma development, treatment with metronidazole and ciprofloxacin resulted in accelerated development of these tumors.^25^ Repeated antibiotic use may also increase risk of certain human malignancies.^26^

## 2 METABOLITES CRITICALLY SHAPE THE MICROBIOME

### 2.1 Dietary micronutrients

Micronutrients being essential food for microbes substantially influence microbiota composition. Most bacteria require iron and have evolved multiple strategies for sequestration including the production of hemophores and low molecular iron chelators named siderophores. Iron supplementation promotes the establishment of bacteria relying on this metal (Table 3). In atopy, a poor iron status is associated with allergy, whereas an improved status abrogates or seems to prevent the onset of allergy. In cancer, iron contributes to tumor growth with high serum iron increasing the risk of several cancers (Table 4).^27^

**Table 3:** Bacterial composition of the microbiota is modified in allergy and depends on the diet

	Phylum	Class	Order	Family	Species
Allergy					
	Firmicutes↑	Clostridia↑	Clostridiales↑	Clostridiaceae↑	Clostridium spp.↑
				Ruminococcaceae↑	Ruminococcus spp.↑
	Bacteroidetes↓	Bacteroidia↓	Bacteroidales↓	Bacteroidaceae↓	Bacteroides spp.↓
Diet					
Ominivore	Firmicutes↑	Clostridia↑	Clostridiales↑	Lachnospiraceae↑	
Oligosacharrides	Actinobacteria↑	Actinobacteria↑	Bifidobacteriales↑	Bifidobacteriaceae↑	Bifidobacterium spp.↑
Western diet/fat	Firmicutes↑	Erysipelotrichia↑	Erysipelotrichales↑	Erysiopelotrichaceae↑	
Vitamin D	Firmicutes↓	Clostridia↓	Clostridiales↓	Lachnospiraceae↓	Coprococcus↓
	Actinobacteria↓	Actinobacteria↓	Bifidobacteriales↓	Bifidobacteriaceae↓	Bifidobacterium spp.↓

**Table 4:** Diet and microbiota metabolites in allergy and cancer

Compound	Structure	Effects in allergy and cancer
Miscellaneous		
Iron	Fe	Poor iron associated with allergy and high serum iron increases risk of several cancers
Folate	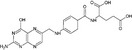	Serum folate is not correlated with allergic reactions, but with serum IgE. High plasma folate decreases risk of wheeze in children
Vitamins		
Vitamin A	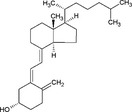	Retinol supplementation has no effect on allergy. Vit A increases Proteobacteria. Increased serum retinol is associated with prostate cancer
Vitamin D	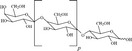	Vitamin D supplementation reduces respiratory infections (not associated with allergy) and possibly reduces asthma incidence in offspring. Vitamin D reduces abundance of Bifidobacterium and Coprococcus and increases Proteobacteria. Vitamin D may prevent hematologic malignancies and advanced colorectal adenomas
Nondigestible oligosaccharides		
Galacto‐oligosaccharides	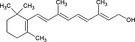	Bacterial fermentation of NDO induces a shift from *Bacteroides* and *Prevotella* species to beneficial *Bifidobacterium* and *Lactobacillus* species Specific NDO feeding reduced the development of AD in infants at risk of allergy, which was associated with increased *Bifidobacterium* and *Lactobacilli* Specific NDO reduce allergic manifestations in experimental models for allergy Specific NDO influence microbiota diversity and prevent the progression of colorectal cancer
Fructooligosaccharides	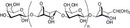	
Agaro‐oligosaccharides	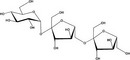	
Short‐chain fatty acids		
Butyrate	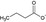	Butyrate and propionate inhibit HDAC activity and promote Treg cells
Propionate	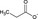	
Acetate	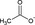	Acetate does not inhibit HDAC

Vitamins A and D (Tables 3 and 4) are important for mucosal immunity.^28^ Retinol supplementation does not change the risk of allergy^29^ or cancer,^30^ though increased serum retinol levels are associated with prostate cancer.^31^ Vitamin D supplementation slightly decreases respiratory infections and the incidence of asthma in offspring,^32^ prevents hematologic malignancies in elderly women and advanced colorectal adenomas,^33^ but seem not to impact allergy risk.^34^ Vitamin D supplementation decreases the abundance of Bifidobacterium and Coprococcus.^35^

Serum folate levels are associated with serum IgE,^36^ high plasma folate is associated with decreased risk of wheezing,^37^ and folic acid supplementation has been linked with an increased risk for lung cancer.^38^ Folate can be produced by most Bacteroidetes, Fusobacteria, and Proteobacteria, but rarely from Actinobacteria and Firmicutes.^39^ In summary, dietary micronutrients shape the microbiome which contributes products essential for an effective immune defense.

### 2.2 Nondigestible Oligosaccharides

Nondigestible oligosaccharides (NDO) are potential substrates for bacterial metabolism in the colon, and a declining microbial diversity in the gut is implicated in the rising incidence of allergic disorders in early life.^40^ Manipulation of the gut microbiota with NDO from natural sources or supplemented galacto‐oligosaccharides (GOS), lactose, and fructooligosaccharides (FOS) holds great promise for the treatment of inflammatory and allergic diseases (Table 4). NDO feeding reduced the development of atopic dermatitis (AD) in infants at risk of allergy which was associated with increased *Bifidobacterium* and *Lactobacilli*,^41^, ^42^ re‐balancing the immune response from a predominant Th2‐type allergic profile at birth to a more Th1‐type and Treg profile. NDO protect against allergic manifestations in experimental models of food allergy and allergic asthma^43^, ^44^ and reshape the gut microbiota with increased levels of short‐chain fatty acids (SCFAs).^45^ Less is known concerning the anti‐cancer effects of NDO.^46^ Experimental models show encouraging effects of GOS, FOS, agaro‐oligosaccharides,^47^ and butyrate^46^ in influencing microbiota diversity (eg, increased Fusobacterium) and the prevention and progression of colorectal cancer.^48^ NDO showed potential in the protection against the development of cancer in healthy subjects.^49^ Since Western diets are typically low on NDO, increasing the intake of NDO may benefit human health via an improved microbiome and/or via direct effects on the immune system.

### 2.3 Microbiota metabolites

Dairy products such as yogurt and butter contain short‐chain fatty acids (SCFAs), while SCFAs are also produced by intestinal bacteria following their fermentation of fibers present in vegetables and fruits.^50^ The major SCFAs are acetate, propionate, and butyrate. SCFAs are an important energy source for intestinal epithelial cells and regulate the assembly and organization of epithelial tight junctions. Abnormalities in the production of these metabolites (due to dietary factors and/or microbial dysbiosis) might play a role in the pathogenesis of type 2 diabetes, obesity, inflammatory bowel disease, colorectal cancer, and allergies.^51^, ^52^ Butyrate influences the activity of histone deacetylases (HDAC), responsible for decreasing dendritic cell IL‐12 and IL‐6 secretion, and allows dendritic cells to promote Tregs. Propionate can also contribute to the induction of T‐cell Foxp3 expression by dendritic cells (DC), while acetate does not have this activity, possibly due to the lack of HDAC activity.^53^ G Protein–coupled receptors GPR109a and GPR43 have been described to contribute to these effects, while GPR43 expression on colonic inducible Treg cells is associated with their expansion and IL‐10 secretion.^54^, ^55^

## 3 MICROBIOTA REGULATING CELLULAR PLAYERS IN INNATE AND ADAPTIVE RESPONSES

### 3.1 Cells of the epithelial barrier

**Figure 1:**
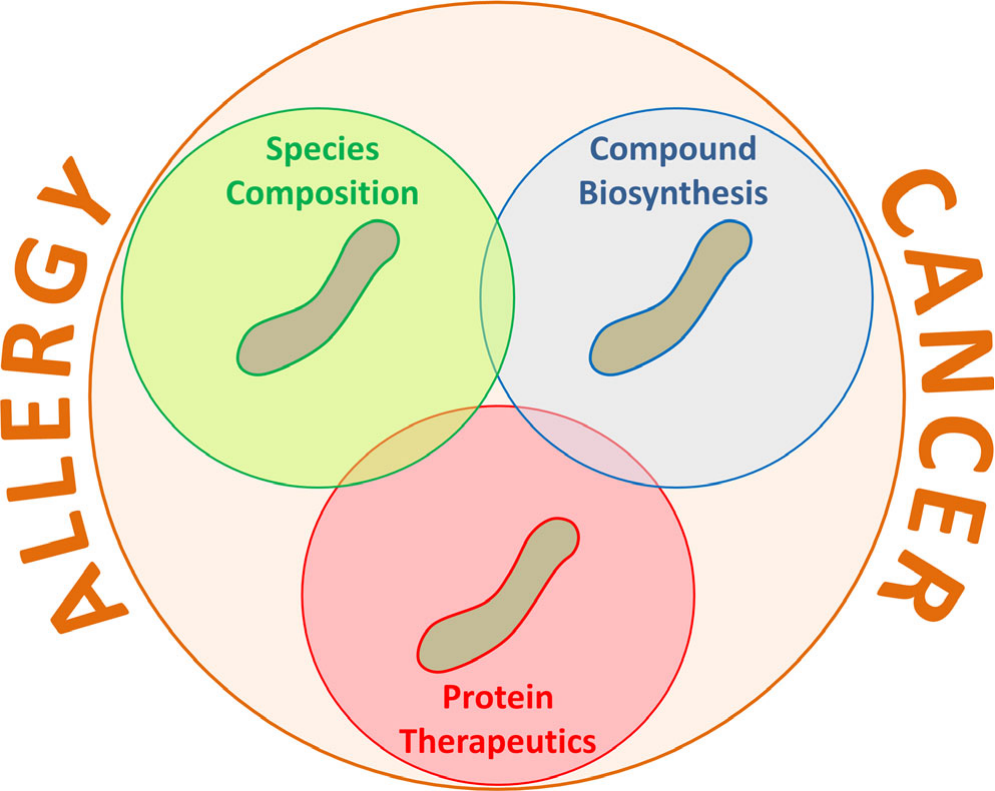
Microbiota engineering. The three main approaches to microbiota engineering are depicted: left, interventions to modify the microbiota species composition; right, engineering biosynthetic pathways for the production of compounds benefiting human health and homeostasis; bottom, engineering selected commensal strains for therapeutic protein live delivery. At the center of each circled approach, a single bacterial cell is depicted as commensal representative. These approaches have possible overlaps that allow better tailored design. All approaches can be envisaged to ameliorate or even prevent both allergy and cancer

Epithelial cells have a very close interaction with compartment‐specific microbiota (Figure 1A). These cells are able to sense conserved microbial‐associated molecular patterns by innate pattern recognition receptors (PRR) such as TLRs and Nod‐like receptors. These interactions can control epithelial cell proliferation and barrier function.^56^ Not only direct interaction, but also microbiota‐derived metabolites such as SCFAs and bacterial quorum sensing (QS) can have an impact on epithelial cells. In the intestine, SCFAs stimulate the inflammasome pathway via binding to GPRs on the surface of epithelial cells. Associated with enhanced epithelial IL‐18 secretion, barrier integrity is enhanced and epithelial cells secrete more antimicrobial peptides.^57^, ^58^ Both, SCFA and QS signals, have immunomodulatory potential by stimulating survival pathways and contributing to intestinal homeostasis.^59^, ^60^, ^61^ Thus, microbiota and metabolites have a paramount influence on epithelial cell function.

### 3.2 Dendritic cells

As professional antigen presenting cells (APC), DC are in contact with both invading pathogens and commensal microbiota and maintain the balance between inflammatory and tolerogenic immune responses. Human monocyte‐derived dendritic cells (moDCs), when matured in the presence of the SCFA butyrate (Figure 1D), increase IL‐10, but decrease IL‐6 and IL‐12 expression.^62^ Similarly, treatment of mice with the SCFA propionate generates new bone marrow DC precursors with high phagocytic capacity but an impaired ability to promote T helper type 2 (Th2) responses in the lung. This effect is dependent on G protein–coupled receptor 41 (GPR41, also called free fatty acid receptor 3 or FFAR3), but not GPR43 (also called free fatty acid receptor 2 or FFAR2).^55^ DCs can also exert roles in adoptive T‐cell therapy (ACT) in cancer (Figure 1E), and the composition of the gut microbiome or treatment with antibiotics could lead to an increase in CD8α^+^ DCs, and consequent IL‐12 release that sustains the anti‐tumor ACT.^63^

### 3.3 Macrophages

The microbiota and its metabolites such as SCFAs strongly influence myelopoiesis and the tissue‐resident macrophages. In the intestine, microbial SCFA butyrate activates its endogenous receptor GPR109a promoting anti‐inflammatory properties in colonic macrophages that induce differentiation of Treg cells and IL‐10‐producing T cells. Such responses may be beneficial in food tolerance, but perhaps not in cancer. Moreover, butyrate exerts an anti‐inflammatory effect on lamina propria macrophages by inhibition of IL‐6, IL‐12, and NO via inhibition of HDACs.^64^ Noteworthy, this mechanism did not affect primary LPS response genes such as *Tnf*α and *Ccl2*. Microbiota can also affect macrophage phenotype and response if they are not in contact with the innate immune system. In the CNS of germ‐free mice, macrophages (microglia) display an altered phenotype. In the lungs of mice treated with antibiotics, macrophages are polarized toward a M2 pro‐allergic phenotype by prostaglandin E2 (PGE_2_) enhanced by commensal fungi.^65^

### 3.4 Mast cells

Mast cells (MC) are crucial effector cells in allergy and other inflammatory diseases.^66^ They also play important roles in regulating the microenvironment of various tumors.^66^ There is increasing evidence that MC function can be modulated by commensal, symbiotic, and pathogenic microorganisms.^67^ Microorganisms may influence MC activation via direct interaction or via secreted metabolites. For example, co‐culture with *Lactobacillus rhamnosus* (Figure 1C) downregulates gene expression for the high‐affinity IgE receptor and histamine H4 receptor in human MC, while increasing IL‐8, IL‐10, CCL2, and TNF‐α.^67^ Stabilization of MC by *L rhamnosus* is possibly induced indirectly via targeting the KCa3.1 channel.^68^ Some Lactobacillus strains, such as *Lactobacillus paracasei*, inhibited IgE‐mediated activation of murine MC with a possible involvement of TLR2. Inhibition by *L casei* is cell contact‐dependent, but TLR‐ or Nod1/2‐independent.^67^ Inhibition of MC activation by microbiota‐derived metabolites such as SCFA may be mediated via the surface receptors GPR41, GPR43, and GPR109A^69^, ^70^ nuclear receptor family peroxisome proliferator‐activated receptors (PPAR) or direct inhibition of histone deacetylase activity.^71^ The bioactive milk peptide glycomacropeptide (GMP) increased intestinal Lactobacillus and Bifidobacterium levels in rats and decreased allergen‐induced MC activation.^72^

### 3.5 Eosinophils

Eosinophils are granulocytic leukocytes that exert important functions in protective immune responses against helminths and other pathogens.^73^ They also serve as key effector cells in allergies and other inflammatory diseases. Recent studies demonstrated that eosinophils crucially participate in maintaining the specific tissue‐resident microbiome.^74^ Conversely, functions of eosinophils are regulated by pathogenic as well as probiotic microorganisms; for example, human eosinophils were found to ingest *Clostridium difficile* (Figure 1B), which subsequently stimulated the release of eosinophil‐derived neurotoxin.^75^ In contrast, ingestion of the probiotic strain *Bifidobacterium bifidum* resulted only in a minor neurotoxin release.^75^ Comparably, mouse eosinophils were found to take up the probiotic *Lactobacillus reuteri*.^76^ Using various mouse models of asthma and AD, probiotics like *Lactobacillus fermentum* and *L rhamnosus* were found to improve allergic inflammation associated with decreased eosinophil infiltration, although a direct effect of probiotic bacterial strains on eosinophils was not demonstrated in these studies.^77^, ^78^

### 3.6 ILCs

Innate lymphoid cells (ILCs) develop normally in the absence of the microbiota; however, signals from commensal microorganisms influence the maturation and acquisition of the tissue‐specific functions of ILCs. ILC3 cells maintain tolerance to commensal‐specific T‐cell responses and their depletion and subsequent abrogation of IL‐22 production, results in loss of intestinal bacteria.^65^ Moreover, ILC3s release GM‐CSF and induce tolerance when intestinal macrophages release IL‐1β in response to microbial sensing. A recent study demonstrates that TNF‐β production by ILC3s is crucial for the production of IgA and for microbiota homeostasis in the intestine. Another ILC subtype, ILC2s, is activated by IL‐25 produced in a microbiota‐dependent manner by epithelial tuft cells. Deletion of the ILC1‐lineage transcription factor T‐bet in the innate immune system results in ILC‐dependent and *Helicobacter typhlonius*‐driven inflammation of the intestine, connected to cancer initiation and promotion.^65^ Taken together, the microbiota and their metabolites shape the spectrum of different immunoregulatory ILCs and modulate their ability to prevent tumor immune evasion or allergic responses.

### 3.7 Tregs

Tregs are potent immune‐regulating cells that play a central role in controlling immune responses (Figure 1A). Tregs can support the reduction of allergic diseases, and, on the other hand, the progression of cancer.^79^ Multiple mechanisms are employed by Treg cells and include production of inhibitory cytokines (IL‐10, TGF‐β, and IL‐35), cytolysis of effector T cells and APCs (via granzymes A and B), direct inhibition of DCs (eg, via PD‐1 and CTLA4) and metabolic disruption of effector cells (CD25, cAMP, adenosine, CD39, and CD73).^80^ Germ‐free mice do not fully develop Tregs, similarly to mice treated with antibiotics or mice lacking Toll‐like receptors (TLRs). However, not all bacterial strains are equally effective in inducing Tregs. *Bifidobacterium longum* 35624, Clostridia, and *Bacteroides fragilis* have been shown to induce intestinal Treg cells, while other bacterial strains do not induce Tregs.^81^, ^82^ Pattern recognition receptor activation on DCs seems to be an important mechanism by which intestinal microbes may promote Treg cell differentiation.^83^

### 3.8 B cells and Bregs

B cells are known to promote allergy through antigen presentation and class‐switching to IgE, whereas under certain conditions, tumor‐infiltrating B cells can be associated with improved patient survival in cancer.^79^, ^84^ In contrast, Breg‐associated immune tolerance can lead to control of allergy or tumor progression in cancer.^79^

The microbiome may have a role in mediating these multifaceted and opposing B‐cell effects in allergy and cancer. Microbiota regulate activation and differentiation of B cells.^85^ Gut microbiota antigens directly trigger B‐cell activation by binding BCRs, and microbial products activate TLR‐expressing B cells, increasing B‐cell survival, antigen presentation, and antibody production.^86^ Microbiota also mediate the release of epithelial cell and eosinophil‐derived cytokines/chemokines that activate and recruit B cells, and may promote T follicular helper cell‐mediated differentiation of plasma cells.^85^ Conversely, the gut microbiome reportedly induces DCs to produce cytokines, such as IL‐1β and IL‐6, which promote B‐cell differentiation to Bregs.^87^

These findings point to a functional cross talk between humoral immunity and the microbiome.

## 4 MOLECULAR CROSS TALK OF MICROBIOTA WITH INNATE AND SPECIFIC IMMUNE DEFENSE

### 4.1 Redox regulation in allergy and cancer

Recent reports demonstrated that certain microbes can stimulate intracellular signaling, involving PTEN, MAPK, and PTP via the generation of reactive oxygen species (ROS) in epithelia, when harboring regulatory redox‐sensitive thiolates.^88^ Commensal bacteria alter the epithelial redox environment by production of oxygen radicals, cause epithelial cell DNA damage, and may harbor carcinogenic properties, for example, in colon carcinoma development.^89^ Furthermore, allergic asthma is associated with an increase in endogenous ROS formation, leading to oxidative stress–induced damage to the respiratory system and weakened antioxidant defenses. These may result in abnormal physiologic function of DNA, proteins, and lipids that can augment bronchial hyperresponsiveness and inflammation.^90^

Overall, the microbiome is implicated in redox regulating pathways, which are relevant for both chronic inflammation and cancer.

### 4.2 Lipocalins

Human lipocalins, such as tear lipocalin 1 and lipocalin 2 present in the nasal mucosa, can interfere with bacterial iron sequestration,^27^ and may directly modulate site‐specific microbial composition. The immunomodulatory and apoptosis‐regulating properties of LCN2 have been linked to its ability to shuttle iron.^91^ In line with the hygiene hypothesis, the limited “microbial exposure” of allergic individuals may also result in deficiencies of the immune regulatory machinery and this can lead to hyper‐reactive responses.^92^ Interestingly, nearly all major mammalian allergens belong to the lipocalin family,^93^ and are similarly able to bind to bacterial and plant‐derived iron chelators, pointing to a potential role for interference of LCN2 function. In several cancer types and in line with a perturbed iron regulation, LCN2 plays an important role in oncogenesis and cancer progression and may serve as a disease biomarker.^94^

### 4.3 Antibodies

Microbiota‐associated antigens can induce IgA‐producing plasma cells in the gut; CD40‐ligand and IL‐21 from T follicular helper cells, APRIL and BAFF from DCs, induce activation‐induced cytidine deaminase (AID) expression by B cells, promoting IgA class switch recombination. As the composition of the microbiota changes, so do the IgA repertoires.^95^

In a recent mouse study, IL‐33‐deficient mice, with significantly lower levels of intestinal IgA and colon‐residing IgA+ B cells, had increased DNA damage‐induced tumors; observations that were ameliorated upon microbiome restoration by co‐housing deficient animals with wild‐type mice.^96^ In humans, altered gut microbiota diversity and low total IgA levels may be associated with the development of allergies and asthma,^97^ and IgA‐deficient individuals have a moderately increased risk of cancer, with higher risks in gastrointestinal cancer.^98^ This disease risk may be a result of impaired mucosal barrier function. However, the associations between microbiota‐driven antibody production with allergies and cancer are not yet sufficiently understood.

## 5 TRANSLATIONAL IMPLICATIONS OF MICROBIOTA

### 5.1 Oncoimmunology and allergy

Specific microbes and the microbiota in general can be considered as important drivers of immunomodulation and can contribute to establishing immune tolerance, with differential impact on the many diverse immune‐mediated diseases. Due to the close interaction and the bidirectional influence of intestinal microbiota on the mucosal immune system, the gut can be considered as an essential site of immune cross talk in the human host. When it comes to allergy or cancer, however, regulation of tolerance has an opposing impact on disease development and treatment.^79^

In cancer, the impact of microbiota was for a long time primarily considered in the context of dysbiosis, increased epithelial translocation and carcinogenic effector mechanisms.^99^ However, in recent years, emerging knowledge points to the role of microbiota, especially in the gut, in anti‐cancer immune mechanisms.^100^ This is due to studies describing a reduced anti‐cancer efficacy of chemotherapeutics such as cyclophosphamide and platinum salts in germ‐free and antibiotic‐treated mice. Moreover, the redox equilibrium of myeloid cells contained in the tumor microenvironment is influenced by intestinal microbiota and due to the importance of the gut in immune fitness, intestinal microbes are essential for the availability of immunomodulators.

In cancer immunotherapy, major advances have been made in the past years to understand the contribution of microbiota composition to successful treatment. Studies are now starting to evaluate the role of the gut microbiome in anti‐PD‐1 immunotherapy,^101^, ^102^ including the first clinical study focusing on PD‐1 checkpoint inhibitor response in metastatic melanoma. In a recent ground‐breaking study, the authors were able to demonstrate a correlation between response to PD‐1 checkpoint inhibitor treatment and diversity of microbial strains in the intestine. Moreover, high abundance of *Faecalibacterium* and low abundance of *Bacteroidales* were associated with good prognosis and longer progression‐free survival after checkpoint inhibitor treatment.^101^ Moreover, toxicity‐related side effects and a dysfunctional intestinal epithelial barrier seem to be attenuated by a beneficial microbiota composition, potentially acting not only via modulation of the host immune response but also via modulation of cancer metabolism.^103^, ^104^

Even though immunotherapy has been the treatment of choice for allergic diseases for more than a century, it seems that the cancer field is more advanced than allergy research with regard to evaluating the role of microbiota. There is a growing number of studies proving the major immunomodulating effects of intestinal microbiota and dietary supplementation with probiotic strains, as well as growing evidence demonstrating the role of intestinal microbial colonization in prevention and onset of allergic disease.^9^, ^105^ However, only a limited number of studies have so far evaluated the influence of beneficial bacterial strains in the context of successful allergen‐specific immunotherapy (AIT). Beneficial immunomodulatory and clinical effects of probiotic supplementation were observed when using different probiotic strains such as *L rhamnosus* in a randomized clinical trial of peanut oral immunotherapy or in grass‐pollen sublingual immunotherapy (SLIT) or by combining SCIT with *Clostridium butyricum* supplementation.^106^, ^107^, ^108^ Thus, major research efforts will be essential in the future to close this current knowledge gap and to ensure optimized AIT formulations for efficient treatment of allergic patients.

### 5.2 Microbial translocation in cancer and allergy

Under certain conditions, the normal gut epithelial barrier can become leaky, permitting the passage of microbes and microbial molecules into the systemic circulation, known as microbial translocation. Microbial products, especially cell walls of gram‐negative bacteria (lipopolysaccharide [LPS] or endotoxin), are powerful stimulators of innate immunity and of B‐cell activation, resulting in inflammation that can lead to disease states, and even septic shock if present in very high doses.^109^ Microbial translocation can be assessed by measuring the immune molecules stimulated by LPS, including soluble CD14 (sCD14), LPS‐binding protein (LBP), and antibodies recognizing the core LPS antigen (Endocab).^109^

Gut barrier permeability is well‐established in the setting of HIV infection. Two different prospective cohort studies reported that immune markers reflecting microbial translocation are associated with an elevated risk of AIDS‐related non‐Hodgkin lymphoma (NHL),^110^, ^111^ presumably due to increased B‐cell activation resulting from the triggered innate inflammation signals. Both studies found that sCD14, measured years prior to diagnosis, was associated with a twofold to fourfold increase in NHL risk, but the findings were inconsistent with respect to the other measures. Besides implications of microbial translocation in food allergy and asthma, it also strongly influences AD.^112^ A randomized trial among adult AD patients, given either selected probiotics or placebo, resulted in clinical improvement, decreased T helper type 2 (Th2) immune activation, and reduced plasma LPS as a measure of microbial translocation.^112^ Thus, microbial translocation may play a role in the etiology of both AIDS‐related lymphoma and AD. It may therefore be possible to decrease risk by reducing microbial translocation through the selective use of probiotics.

### 5.3 Outlook: microbiota engineering

**Figure 2:**
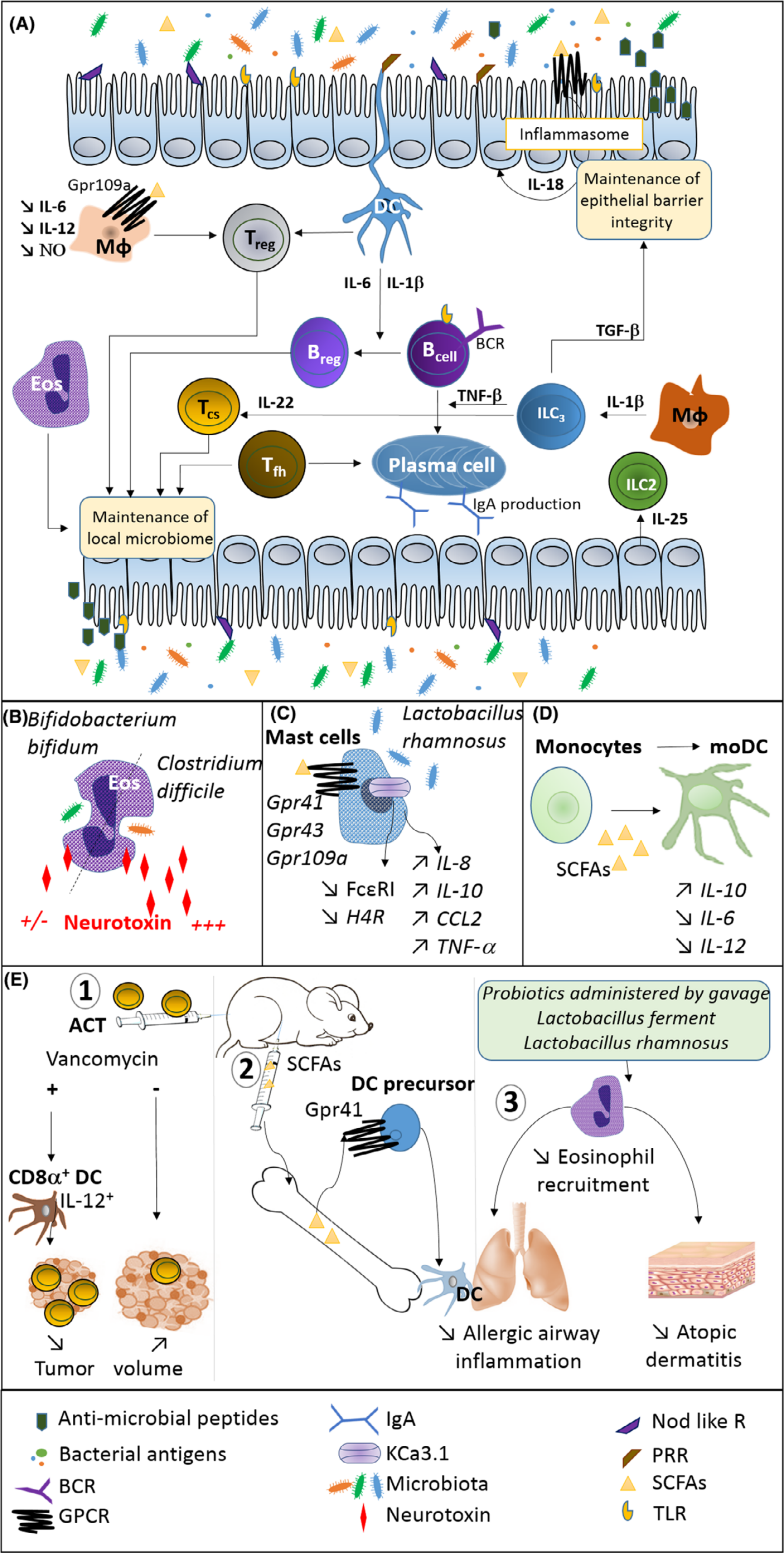
Interaction of microbiota with immune cells in health and disease. A, Bidirectional interaction between gut microbiome and players of the immune system. Cells from the epithelial barrier (epithelial cells and DC) sense the microbiome through the expression of a large panel of receptors. In health, such interactions are essential for the maintenance of the local microbial homeostasis and the integrity of epithelial barriers. This mechanism involves a variety of regulatory immune cells resident in the gut mucosa as well as molecules secreted by the epithelial layer. B, Human eosinophils were shown to secrete, among others, high amounts of neurotoxin following phagocytosis of the pathogenic bacteria Clostridium difficile that were limited following the engulfment of Bifidobacterium bifidum. C, In vitro interaction of human mast cells with Lactobacillus rhamnosus and SCFAs results in their modified functions and phenotypes with the implication of the KCa3.1 channel as well as Gpr41, 43, and 109a, respectively. D, The differentiation of human monocytes into moDC in the presence of SCFAs induces modified secretory capacities compared to controls. E, The efficacy of adoptive T‐cell therapy was proven to be associated with the microbiome. The success of adoptive T‐cell therapy correlates with a peripheral increase and a more abundant tumor infiltration of CD8α^+^ DC producing IL‐12.^63^ Mouse treatment with SCFA results in the recruitment of bone marrow‐derived DC with impaired capacity to induce Th2 responses in the lung.^55^ Probiotics administered by gavage reduce the severity of allergic airway inflammation and AD through the reduction of eosinophil infiltration into the lung and skin, respectively^77^, ^78^

The beneficial effect of a balanced microbiota on human health can be restored or potentiated by external/medical intervention. There are three main routes to microbiota engineering (Figure 2). Firstly, in pathological conditions, commensal bacteria could be administered to move toward a species composition more representative of a healthy microbiota. Examples are fecal transplantation and vaginal swab, but other types of more specific interventions are being studied. Secondly, bacterial biosynthetic pathways could be engineered for de novo or enhanced production of compounds that can promote healthier mucosal environments and homeostasis. Thirdly, selected commensal bacterial strains (especially lactic acid bacteria) could be engineered for the live delivery of recombinant therapeutic proteins either to prevent or to combat disease.^113^, ^114^ To this end, live recombinant protein delivery could occur via secretion, membrane‐anchorage, or intracellular load. Engineering biosynthetic pathways or exogenous recombinant protein production implies the genetic modification of commensal bacteria; therefore, sophisticated strategies to control their survival in the host and in the local environment are being devised.^115^ Cancer and allergy are widely the focus of many of these efforts (Figure 2), with a number of studies showing how microbiota composition and its engineering may ameliorate or support the appropriate immune response to contribute to clinical benefits for each condition.^116^

## 6 CONCLUSION

The biodiversity hypothesis is a cornerstone in the understanding of the allergy epidemics. Subsequently, the era of microbiota research has opened up novel perspectives on allergy pathogenesis,^117^ but also on cancer due to the immunomodulatory properties of the mutualistic microbes. The impact of the microbiota can be direct or indirect by cellular cross talk with and among innate or specific immune cells, by translocation of microbiota via epithelia into tissues, or by the exchange of molecules which may stimulate inflammatory or regulatory cells. The subsequent result can be inflammation or reconstitution, with opposing consequences in allergy and cancer. Importantly, the composition of the human microbiota can be disturbed by environmental factors, composition of the diet, and especially by medical interventions such as antibiotics and anti‐ulcer medications. Phylogenetic variability and stable composition of microbiota seem to be determined in early life, defining this as a critical period for establishing health and homeostasis. However, it also offers a potential window of opportunity for interventions aiming at the establishment of a healthy microbiota to support long‐term human health.

## CONFLICTS OF INTEREST

Dr Erika Jensen‐Jarolim is shareholder of Biomedical International R+D, Vienna, Austria. Dr Sophia N. Karagiannis is a founder and shareholder of IGEM Therapeutics Ltd. Dr Liam O'Mahony reports personal fees from Alimentary Health Ltd, grants from GSK, outside the submitted work. Dr Manuel L. Penichet is a shareholder of Klyss Biotech, Inc. The Regents of the University of California licensed Dr Penichet's technology to this firm. All other authors declare no conflicts of interest in relation to this publication.

## AUTHOR CONTRIBUTIONS

UE drafted the manuscript, contributed “Cells of the epithelial barrier,” “Oncoimmunology and allergy” and Table 1, compiled all contributions, and contributed to the Abstract and Introduction; BHJ and KSN contributed “B cells and Bregs,” and with GHJ “antibodies”; BC “Redox regulation in allergy and cancer”; BR contributed “DC,” “Macrophages,” and “ILCs”; CW and PM contributed “Microbial translocation”; RF composed “Nondigestible Oligosaccharides” and Table 4; HK and LSF contributed “Eosinophils”; RF with HK contributed “Mast cells”; OML contributed chapters “Microbiota metabolites” and “Tregs”; RWF wrote “Lipocalins”, “Dietary micronutrients,” and Tables 2 and 3; JDH contributed “Hygiene hypothesis in cancer” and Table S1; VL contributed “Microbiota engineering” and designed Figure 2; JJE and TMC wrote “Hygiene hypothesis in allergy” with Table S1. PA designed Figure 1; PA, BHJ, and JDH completed the graphical abstract based on the paper contents; JJE and KSN orchestrated the whole process, wrote the Conclusion and Abstract, and conducted the final editing.
