# Supplementary material for: AllergoOncology: Microbiota in allergy and cancer—A European Academy for Allergy and Clinical Immunology position paper
Source: Allergy. 2019 Mar 6;74(6):1037–51. doi: 10.1111/all.13718 (PMC6563061; doi:10.1111/all.13718)
Supplement: Supplementary file 1 [file ALL-74-1037-s001.docx]

**Supplementary Material**

**Suppl. Table 1. Analysing the farm effect protective against allergy and asthma.**

| **Topic** | **Evidence** | **Reference** |
| --- | --- | --- |
| Allergy is epidemic | Review | Platts Mills (1) |
| Farms protect against allergy epidemic | Review | Von Mutius (2) |
| Farm effect is related to microbiome | Review | Ege (3) |
| Farms effects in utero and early childhood protect against atopy | Epidemiology:1746 farmers and 1555 spouses at US farms. Maternal farming in pregnancy: adjusted OR = 0.60; 95% CI, 0.48-0.74 | House et al (4) |
| Farm milk protects against viruses | Epidemiology: 983 infants from Middle Europe. Risk for infections reduced by 30%. Raw milk consumption inversely associated with rhinitis (adjusted OR = 0.71 [0.54-0.94]), respiratory tract infections (0.77 [0.59-0.99]), otitis (0.14 [0.05-0.42]), and fever (0.69 [0.47-1.01]) | Loss et al (5), Ludka et al (6) |
| Farm dust levels in geocoded areas | Proximity to farm <100m is protective | Müller et al (7) |
| Amish farms protect better | Review; | Ober et al (8) |
|  | Original data: allergic sensitization 4 and 6 times lower in Amish, supported by mouse model | Stein et al (9) |
| Cohabitation protects mice | Review | Reardon (10) |
| Cohabitation protects mice | Review | Gupta (11) |
| **Protective Factors** | | |
| Helminths support microflora | Review; | Cruz et al (12); |
|  | Mouse helminth infection model | Ramandan et al (13) |
| Viral influenza peptides | 2 Mouse models and in vitro studies | Skevaki et al (14) |
| Non-bacterial compound: sialic acid N-glycolylneuraminic acid (Neu5Gc) | Anti-Neu5Gc IgG levels and Tregs correlated positively with living on a farm; Neu5Gc antiinflammatory in mouse models. Epidemiology, serology from 2 studies: 1.) from PARSIFAL study^1^ (n = 299), 2.) from the PASTURE^2^ birth cohort (cord blood [n = 836], 1 year [n = 734], 4.5 years [n = 700], and 6 years [n = 728]). | Frei et al (15) |
| Bacterial LPS and ubiquitin-modifying enzyme A20 | 1.) LPS and dairy stable dust protected transgenic Tnfaip3^EC-WT^, but not of Tnfaip3^EC-KO^ mice from HDM-induced asthma; 2.) LPS and dairy stable dust *in vitro* reduced GM-CSF and IL-1α production upon HDM stimulation from human endobronchial biopsies; 3.) GABRIELA ^3^ study samples: the protective effect of growing up on farm against asthma stronger in children with the G allele of *Tnfaip3* coding for A20 [adjusted OR = 0.14 (0.05 to 0.39)] | Schujis et al (16) |
| **Protective mechanisms** | | |
| Farm dust and Endotoxin on bronchial epithelia counteract Th2 immunity | Gene-by-environment interaction: A single-nucleotide polymorphism results in a G-allele of the *TNFAIP3* gene encoding for A20 is responsible for the protective effect (see also section above) | Schujis et al (16) |
| Farm milk exposure induces Tregs and FOXP3 demethylation | 298 children from PASTURE birth cohort study were included in this study (149 farm and 149 reference children): Elevated Tregs were significantly associated with asthma: odds ratio, 0.26; 95% CI, 0.08-0.88) and perennial IgE (odds ratio, 0.21; 95% CI, 0.08-0.59). | LLuis et al (17), |
| Maternal farm exposure induces Tregs and Th17 response | 84 pregnant mothers: A positive correlation between TH17 lineage markers and FOXP3 (mRNA), influenced by maternal farming | LLuis et al (18) |
| Enhancement of epithelial barrier | CpG (1 or 10 μmol/L) enhances bronchial integrity in vitro; | Kubo et al (19), |
|  | Farm dust in vitro enhanced barrier function and antiviral response | Perdijk et al (20) |
|  | Review: Puts epithelial barrier in focus of correlation of microbiome dysregulation and allergy | Lambrecht and Hammad (21), |
|  |  | Vlugt et al (22) |

1…PARSIFAL - Prevention of Allergy Risk factors for Sensitization in Children Related to Farming and Anthroposophic Lifestyle

2…PASTURE - Protection Against Allergy Study in Rural Environments

3…GABRIELA – Multidisciplinary Study to Identify the Genetic and Environmental Causes of Asthma in the European Community [GABRIEL] Advanced Study

**References to Suppl. Table 1**

1. Platts-Mills TA. The allergy epidemics: 1870-2010. *J Allergy Clin Immunol* 2015;**136**(1):3-13.

2. von Mutius E. The microbial environment and its influence on asthma prevention in early life. *J Allergy Clin Immunol* 2016;**137**(3):680-689.

3. Ege MJ. The Hygiene Hypothesis in the Age of the Microbiome. *Ann Am Thorac Soc* 2017;**14**(Supplement_5):S348-S353.

4. House JS, Wyss AB, Hoppin JA, Richards M, Long S, Umbach DM, et al. Early-life farm exposures and adult asthma and atopy in the Agricultural Lung Health Study. *J Allergy Clin Immunol* 2017;**140**(1):249-256 e214.

5. Loss G, Depner M, Ulfman LH, van Neerven RJ, Hose AJ, Genuneit J, et al. Consumption of unprocessed cow's milk protects infants from common respiratory infections. *J Allergy Clin Immunol* 2015;**135**(1):56-62.

6. Ludka-Gaulke T, Ghera P, Waring SC, Keifer M, Seroogy C, Gern JE, et al. Farm exposure in early childhood is associated with a lower risk of severe respiratory illnesses. *J Allergy Clin Immunol* 2018;**141**(1):454-456 e454.

7. Muller-Rompa SEK, Markevych I, Hose AJ, Loss G, Wouters IM, Genuneit J, et al. An approach to the asthma-protective farm effect by geocoding: Good farms and better farms. *Pediatr Allergy Immunol* 2018.

8. Ober C, Sperling AI, von Mutius E, Vercelli D. Immune development and environment: lessons from Amish and Hutterite children. *Curr Opin Immunol* 2017;**48**:51-60.

9. Stein MM, Hrusch CL, Gozdz J, Igartua C, Pivniouk V, Murray SE, et al. Innate Immunity and Asthma Risk in Amish and Hutterite Farm Children. *N Engl J Med* 2016;**375**(5):411-421.

10. Reardon S. Dirty room-mates make lab mice more useful. *Nature* 2016;**532**(7599):294-295.

11. Gupta S. Microbiome: Puppy power. *Nature* 2017;**543**(7647):S48-S49.

12. Cruz AA, Cooper PJ, Figueiredo CA, Alcantara-Neves NM, Rodrigues LC, Barreto ML. Global issues in allergy and immunology: Parasitic infections and allergy. *J Allergy Clin Immunol* 2017;**140**(5):1217-1228.

13. Ramanan D, Bowcutt R, Lee SC, Tang MS, Kurtz ZD, Ding Y, et al. Helminth infection promotes colonization resistance via type 2 immunity. *Science* 2016;**352**(6285):608-612.

14. Skevaki C, Hudemann C, Matrosovich M, Mobs C, Paul S, Wachtendorf A, et al. Influenza-derived peptides cross-react with allergens and provide asthma protection. *J Allergy Clin Immunol* 2017.

15. Frei R, Ferstl R, Roduit C, Ziegler M, Schiavi E, Barcik W, et al. Exposure to nonmicrobial N-glycolylneuraminic acid protects farmers' children against airway inflammation and colitis. *J Allergy Clin Immunol* 2018;**141**(1):382-390 e387.

16. Schuijs MJ, Willart MA, Vergote K, Gras D, Deswarte K, Ege MJ, et al. Farm dust and endotoxin protect against allergy through A20 induction in lung epithelial cells. *Science* 2015;**349**(6252):1106-1110.

17. Lluis A, Depner M, Gaugler B, Saas P, Casaca VI, Raedler D, et al. Increased regulatory T-cell numbers are associated with farm milk exposure and lower atopic sensitization and asthma in childhood. *J Allergy Clin Immunol* 2014;**133**(2):551-559.

18. Lluis A, Ballenberger N, Illi S, Schieck M, Kabesch M, Illig T, et al. Regulation of TH17 markers early in life through maternal farm exposure. *J Allergy Clin Immunol* 2014;**133**(3):864-871.

19. Kubo T, Wawrzyniak P, Morita H, Sugita K, Wanke K, Kast JI, et al. CpG-DNA enhances the tight junction integrity of the bronchial epithelial cell barrier. *J Allergy Clin Immunol* 2015;**136**(5):1413-1416 e1411-1418.

20. Perdijk O, van Splunter M, Savelkoul HFJ, Brugman S, van Neerven RJJ. Cow's Milk and Immune Function in the Respiratory Tract: Potential Mechanisms. *Front Immunol* 2018;**9**:143.

21. Lambrecht BN, Hammad H. The immunology of the allergy epidemic and the hygiene hypothesis. *Nat Immunol* 2017;**18**(10):1076-1083.

22. van der Vlugt L, Eger K, Muller C, Ninaber DK, Zarcone MC, Amatngalim GD, et al. Farm dust reduces viral load in human bronchial epithelial cells by increasing barrier function and antiviral responses. *J Allergy Clin Immunol* 2018.
